# Editorial: Artificial intelligence applications for cancer diagnosis in radiology

**DOI:** 10.3389/fradi.2025.1493783

**Published:** 2025-01-29

**Authors:** Abhirup Banerjee, Hongming Shan, Ruibin Feng

**Affiliations:** ^1^ Institute of Biomedical Engineering, Department of Engineering Science, University of Oxford, Oxford, United Kingdom; ^2^ Division of Cardiovascular Medicine, Radcliffe Department of Medicine, University of Oxford, Oxford, United Kingdom; ^3^ Institute of Science and Technology for Brain-Inspired Intelligence, MOE Frontiers Center for Brain Science, and MOE Key Laboratory of Computational Neuroscience and Brain-Inspired Intelligence, Fudan University, Shanghai, China; ^4^ Stanford Cardiovascular Institute, Stanford University, Stanford, CA, United States

**Keywords:** artificial intelligence, cancer detection, computer vision, deep learning, interpretability, machine learning, medical image analysis, radiology

**Editorial on the Research Topic**
Artificial intelligence applications for cancer diagnosis in radiology

Cancer remains one of the most significant threats to human life, with early detection being particularly challenging. Radiological imaging is a primary tool in identifying cancers, yet the early signs are often subtle, leading to potentially treatable cancers being missed (1). Artificial intelligence (AI) holds immense promise as a powerful tool to assist radiologists in cancer detection (2). AI algorithms have demonstrated impressive capabilities in cancer identification, segmentation, and assessment (3, 4). However, the opaque nature of these algorithms—often referred to as their “black-box” characteristics—raises concerns about their interpretability and the verifiability of their clinical predictions (5). Several emerging challenges need to be addressed to effectively integrate AI into cancer detection. During data curation, the publicly available datasets are often limited by small size, incomplete labelling, or variability in scanner technology and imaging protocols, which restricts their applicability (6). In the development phase, AI algorithms depend heavily on manual annotations from expert radiologists, and their performance may decline when applied to data from different hospitals or protocols (7). Furthermore, current AI models struggle with issues such as handling partial or noisy labels, managing long-tailed data distributions, and adapting to continual learning (8).

**Figure 1:**
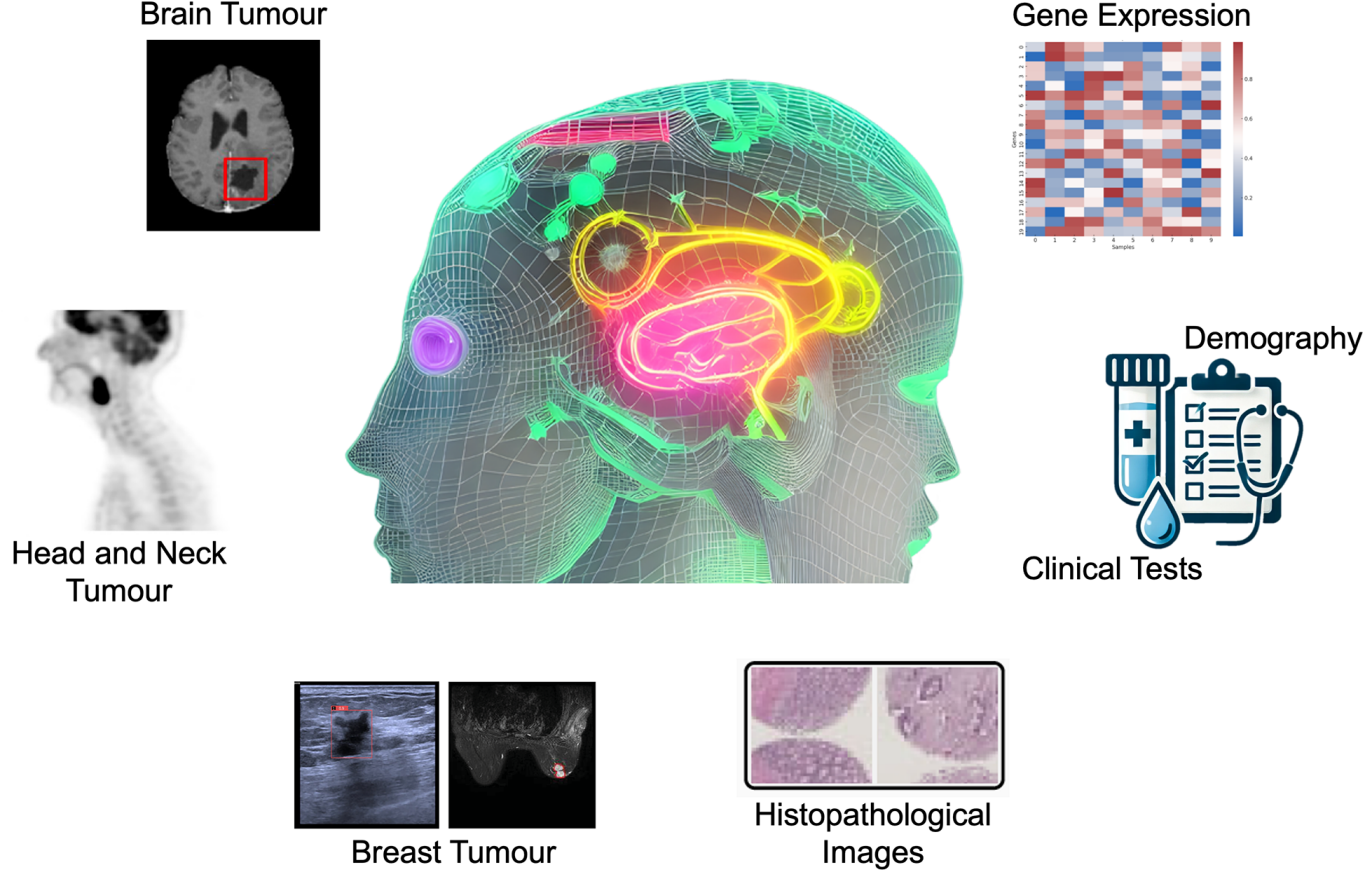
Artificial intelligence applications for cancer diagnosis in radiology.

To enhance the clinical adoption of AI as a reliable and user-friendly tool, it is necessary to develop AI systems that can work synergistically with radiologists, combining the strengths of human expertise and AI to improve cancer detection and patient outcomes (9). This Research Topic has curated articles on the applications of AI models, especially the machine learning models of Random Forest (RF), Neural Networks (NN), Bootstrap Aggregating Classification and Regression Trees (Bagged CART), Extreme Gradient Boosting Tree (XGBoost), and elastic net, and deep learning models of convolutional neural network (CNN), U-Net, ResNet, and multi-head attention fusion, for the tasks of brain tumour segmentation (Luque et al.), head and neck tumours segmentation (Zhang and Ray), breast cancer subtype classification (Sun et al.) and risk factors identification (Dianati-Nasab et al.), incidentally discovered breast mass classification (Ma et al.), and rectal cancer survival risk prediction (Shu et al., Xu et al.), using different imaging modalities including Magnetic Resonance Imaging (MRI), Positron Emission Tomography (PET), ultrasound, as well as transcriptomic, clinical, lifestyle, and sociodemographic data (Figure 1).

Luque et al. developed a U-Net-based deep-learning model to segment contrast-enhancing glioblastoma tumours on early post-operative MRI scans and classify the extent of resection (EOR) as maximal or submaximal. Trained on 122 multiparametric MRI scans, the model achieved a mean Dice score of 0.52 ± 0.03 on an external dataset (*n* = 248), comparable to expert interrater agreement. It demonstrated precision/recall scores of 0.72/0.78 on an internal test dataset (*n* = 462) and 0.90/0.87 on the external dataset. Kaplan-Meier curves showed no significant difference in overall survival predictions between clinical and model-based EOR classifications. This demonstrates that the model effectively classifies EOR and offers prognostic value on par with traditional clinical methods, potentially enhancing patient stratification in glioblastoma treatment.

Accurate tumour segmentation is crucial for effective radiotherapy planning, especially with advanced methods like intensity modulated radiation therapy dose painting, which requires precise delineation of multiple intensity contours for optimal dose distribution. Automated 3D image segmentation using CNNs often struggle with precise boundary identification due to information loss in downsampling layers. In order to address this challenge, Zhang and Ray proposed a novel 3D coarse-to-fine framework, KsPC-Net, combining a CNN with a kernel smoothing-based probability volume contour (KsPC) approach, for segmenting head and neck tumours in 3D PET images. KsPC-Net generates accurate probability contours and object boundaries essential for dose painting strategies with its CNN backbone learning kernel smoothing parameters automatically. Tested against the MICCAI 2021 challenge dataset (HECKTOR), KsPC-Net outperforms existing models, demonstrating its efficacy in improving radiotherapy planning precision.

Sun et al. introduced CAMBNET, a novel deep learning model using cross-attention multi-branch CNN in order to classify luminal and non-luminal breast cancer subtypes using dynamic contrast-enhanced MRI. The model was tested on 160 cases of invasive breast cancer, incorporating patient-specific factors like nodule size and age at menarche. CAMBNET outperformed several classical deep learning models, achieving high diagnostic performance metrics, including an accuracy of 88.44% and an Area Under the Curve (AUC) of 96.10%. Specifically, it showed enhanced accuracy in classifying subtypes for patients with menarche at age 14, where it reached an AUC of 99.95%. The study demonstrates CAMBNET as a promising tool for improving molecular subtype classification, potentially leading to better prognosis and survival outcomes in breast cancer patients.

Aiming to enhance breast cancer prevention and management, Dianati-Nasab et al. conducted a large case-control study to investigate the use of machine learning models for identification of risk factors for primary invasive breast cancer in an Iranian population. The study analysed a dataset of 1,009 cases and 1,009 controls, encompassing lifestyle, health-behaviour, reproductive, and sociodemographic factors. Machine learning models including RF, NN, Bagged CART, and XGBoost were employed. Key predictors of breast cancer identified were a history of chest x-rays, deliberate weight loss, abortion history, and post-menopausal status, along with second-hand smoking, lower education, menarche age (>14), employment, first delivery age (18–23), and breastfeeding duration (>42 months). RF demonstrated the highest performance with an AUC of 0.9 and an accuracy of 83.9%, while XGBoost and NN models showed lower AUC and accuracy. These findings could inform targeted preventive strategies for high-risk women.

Ma et al. investigated the impact of off-the-shelf AI software on classifying incidentally discovered breast masses via ultrasound, addressing issues of inconsistent diagnoses and unnecessary biopsies. Conducted across two health centres from May 2021 to May 2023, the study involved 196 patients with 202 breast masses, categorised using the 5th edition of the Breast Imaging Reporting and Data System (BI-RADS). Pathological results from biopsies or surgeries were used as the gold standard. AI assistance in BI-RADS classification was compared with assessments by two junior and one senior radiologist using receiver operating characteristic (ROC) curves. Results showed AI improved the accuracy, sensitivity, and negative predictive value for junior radiologists, aligning their performance with that of experienced radiologists. AI particularly enhanced diagnostic efficiency for BI-RADS 4a and 4b masses, reducing unnecessary repeat exams and biopsies, thus optimising resource use and diagnostic effectiveness.

Shu et al. developed and validated a prognostic risk prediction model based on immune-related genes (IRGPM) to predict disease-free survival (DFS) in patients with locally advanced rectal cancer (LARC) undergoing neoadjuvant chemoradiotherapy. Using transcriptomic and clinical data from the Gene Expression Omnibus (GEO) and West China Hospital, the study employed the elastic net method to identify key immune-related genes impacting DFS. The IRGPM, constructed using RF techniques, categorised patients into high-risk and low-risk groups based on prognostic risk scores. Analysis of 407 LARC samples revealed a signature of 20 immune-related genes. Kaplan-Meier survival analysis and ROC curves confirmed the model's predictive accuracy. Validation in independent cohorts showed significant differences in immune profiles between risk groups, with the low-risk group exhibiting higher immune activation, including increased levels of activated B cells, CD8 T cells, macrophages, and elevated PDCD1 expression. The IRGPM effectively distinguishes DFS among LARC patients, highlighting its potential for guiding treatment strategies.

Using both digital histopathological images and non-imaging clinical data, Xu et al. presented a multi-modal deep learning framework to forecast the survival of rectal cancer patients. The study considered 292 patients diagnosed between January 2015 and December 2016, splitting them into 234 training and 58 testing cases. Digital pathology images from tissue microarrays and clinical data were pre-processed and used in survival prediction models. Individual deep learning models predicted survival from histopathological images, with the modified ResNet model achieving an AUC of 0.797. A multi-head attention fusion model combining both image and clinical features improved prediction accuracy, reaching an AUC of 0.837 for overall survival. The study demonstrates that integrating digital pathology with clinical data enhances survival prediction, providing valuable insights for clinical practice.

In summary, this Research Topic highlights the potential of integrating AI into cancer detection and prognosis, emphasising the benefits of multi-modal machine learning and deep learning models. AI has shown promise in enhancing the accuracy of tumour detection and classification across various cancer types, such as glioblastoma, head and neck, breast, and rectal cancers, using imaging and clinical data. Despite challenges like data variability and the need for model interpretability, the studies demonstrate that AI can bridge performance gaps between junior and senior radiologists, improve diagnostic precision, and offer robust prognostic tools. To address the “black-box” nature of AI, future efforts should prioritise the development and adoption of explainable AI (XAI) frameworks. These frameworks leverage techniques such as saliency maps, feature attribution methods, and surrogate models to offer clinicians clearer visual or conceptual insights into AI decision-making processes. For example, the integration of XAI into breast cancer diagnostics has demonstrated its potential to enhance trust and transparency, fostering greater clinician understanding and confidence in AI-driven recommendations (10). Additionally, embedding domain knowledge into AI systems—such as aligning them with established medical guidelines or clinical rules—can further bolster their interpretability and reliability. Future research must also address the challenges posed by data variability through the standardisation of datasets and protocols, a critical step to ensure consistency and reproducibility in AI model development (11). Federated learning models present a promising avenue, enabling privacy-preserving collaborations across multiple institutions, which is vital for expanding data diversity while safeguarding patient confidentiality. Furthermore, refining AI algorithms to seamlessly integrate into real-world clinical workflows is essential to their practical adoption. By coupling advancements in explainability with efforts to enhance usability, AI systems can be more effectively integrated into clinical practice. Such advancements hold the potential to revolutionise cancer diagnostics and treatment, paving the way for personalised care strategies and significantly improved patient outcomes.
